# Predicting nutrition and environmental factors associated with female reproductive disorders using a knowledge graph and random forests

**DOI:** 10.1016/j.ijmedinf.2024.105461

**Published:** 2024-04-17

**Authors:** Lauren E Chan, Elena Casiraghi, Justin Reese, Quaker E. Harmon, Kevin Schaper, Harshad Hegde, Giorgio Valentini, Charles Schmitt, Alison Motsinger-Reif, Janet E Hall, Christopher J Mungall, Peter N Robinson, Melissa A Haendel

**Affiliations:** a Oregon State University, College of Public Health and Human Sciences, Corvallis, OR, USA; b AnacletoLab, Dipartimento di Informatica, Università degli Studi di Milano, Italy; c Environmental Genomics and Systems Biology Division, Lawrence Berkeley National Laboratory, Berkeley, CA, USA; d European Laboratory for Learning and Intelligent Systems, ELLIS; e National Institute of Environmental Health Sciences, Epidemiology Branch, Durham, NC, USA; f University of Colorado Anschutz Medical Campus, Aurora, CO, USA; g National Institute of Environmental Health Sciences, Office of Data Science, Durham, NC, USA; h National Institute of Environmental Health Sciences, Biostatistics & Computational Biology Branch, Durham, NC, USA; i National Institute of Environmental Health Sciences, Clinical Research Branch, Durham, NC, USA; j The Jackson Laboratory for Genomic Medicine, 10 Discovery Drive, Farmington, CT, USA; k University of North Carolina, Dept. of Genetics, Chapel Hill, NC, USA

**Keywords:** Female reproductive disorders, Knowledge graph, Endometriosis, Ovarian cysts, Ontologies, Random forest

## Abstract

**Objective:**

Female reproductive disorders (FRDs) are common health conditions that may present with significant symptoms. Diet and environment are potential areas for FRD interventions. We utilized a knowledge graph (KG) method to predict factors associated with common FRDs (for example, endometriosis, ovarian cyst, and uterine fibroids).

**Materials and Methods:**

We harmonized survey data from the Personalized Environment and Genes Study (PEGS) on internal and external environmental exposures and health conditions with biomedical ontology content. We merged the harmonized data and ontologies with supplemental nutrient and agricultural chemical data to create a KG. We analyzed the KG by embedding edges and applying a random forest for edge prediction to identify variables potentially associated with FRDs. We also conducted logistic regression analysis for comparison.

**Results:**

Across 9765 PEGS respondents, the KG analysis resulted in 8535 significant or suggestive predicted links between FRDs and chemicals, phenotypes, and diseases. Amongst these links, 32 were exact matches when compared with the logistic regression results, including comorbidities, medications, foods, and occupational exposures.

**Discussion:**

Mechanistic underpinnings of predicted links documented in the literature may support some of our findings. Our KG methods are useful for predicting possible associations in large, survey-based datasets with added information on directionality and magnitude of effect from logistic regression. These results should not be construed as causal but can support hypothesis generation.

**Conclusion:**

This investigation enabled the generation of hypotheses on a variety of potential links between FRDs and exposures. Future investigations should prospectively evaluate the variables hypothesized to impact FRDs.

## 1. Introduction

Female reproductive disorders (FRDs) such as endometriosis, uterine fibroids, and ovarian cysts significantly affect physical and emotional health, disability, and fertility for women and those assigned female at birth [1]. FRDs fall into a category of conditions that are often misdiagnosed and have prolonged diagnostic timeframes and limited therapeutic options [2,3]. Prevalence of common FRDs such as endometriosis is often underestimated given the clinical difficulty of identifying the condition without invasive laparoscopic surgery and the often years-long lag between symptom onset and diagnosis [2,4]. Due to their widespread prevalence and substantial impact on daily life, ways to more easily identify FRDs as well as viable therapeutic approaches for FRDs are highly sought after [5–7]. Diet and environment have been proposed as potential intervention opportunities for FRDs [8,9], but standard clinical recommendations on diet and exposures are limited. Focusing on modifiable features such as diet, lifestyle factors, and environmental exposures may offer new options for individuals and care providers to manage these common conditions and improve outcomes. We present an innovative approach for assessing survey-based data to predict links between nutrition, environmental exposures, comorbidity, and medication and three common FRDs, namely endometriosis, uterine fibroids, and ovarian cysts.

### 1.1. Common FRDs

Endometriosis is the extrauterine growth of endometrial tissue (also called lesions) with hallmark symptoms that include pelvic pain, dysuria, dysmenorrhea, and sub- or infertility [10]. This FRD is estimated to occur in 10 % of women [11]. Delays in diagnosis are common with endometriosis, and many individuals wait years for a conclusive diagnosis [2,4]. Accordingly, estimates of prevalence vary widely and are likely inaccurate. An estimated 35–50 % of individuals diagnosed with endometriosis experience pain and/or infertility [5], but approximately 20–25 % of individuals with endometriosis do not experience pelvic pain [5,12,13]. Because symptoms can be inconsistent, clinical diagnosis is difficult. Endometriosis is often diagnosed during treatment for fertility issues [14,15]. Endometriosis can present similarly to other gynecological disorders including primary dysmenorrhea, pelvic inflammatory disease, and pelvic adhesions presenting as chronic pelvic pain, painful menses, tubal pregnancies, and infertility [2,3]. Due to its inconsistent presentation, surgical visualization is needed to definitively diagnose endometriosis, which is a barrier to diagnosis and treatment [2].

Uterine fibroids, also called leiomyomas, are common benign tumors estimated to be present in 70–80 % of women by the age of menopause, [16] and approximately 20–25 % of those individuals present with clinical symptoms [17]. The fibroids are composed of smooth muscle cells and fibrous extracellular matrix that is overproduced and creates tumors within the myometrium [18]. Many women with fibroids are not clinically diagnosed. Some have no symptoms, and some live with significantly burdensome symptoms without a clinical diagnosis. The high prevalence of undiagnosed fibroids means that prevalence may be underestimated when determined using clinical records. Common fibroid symptoms include heavy menses, pelvic pain, anemia, urinary incontinence, and infertility [18–20]. With symptomatic fibroids, pregnancy complications (placenta previa, intrauterine growth restriction, increased need for cesarean section) can be more common [21]. Diagnosis of fibroids is usually accomplished with a variety of imaging techniques, including transvaginal ultrasound, hysterosalpingography, saline infusion sonography, hysteroscopy, and magnetic resonance imaging (MRI) [21–23].

Ovarian cysts affect approximately one in 25 women [7]. There are multiple types of ovarian cysts, but functional cysts are the most prevalent. Functional cysts occur when a follicle forms in the ovary, but no ovulation ensues and the follicle does not rupture, creating a cyst [24]. The most frequently reported symptoms of ovarian cysts are pelvic pain, abdominal pressure, bloating, and infertility although asymptomatic ovarian cysts can occur [25,26]. Asymptomatic ovarian cysts can be left untreated and may not require intervention, with some cysts disappearing naturally. However, cysts affecting fertility, pelvic anatomy, or quality of life in a significant way can be surgically removed [27]. While polycystic ovary syndrome (PCOS) is a condition that includes the presence of ovarian cysts, this investigation does not include PCOS as a primary outcome of interest.

### 1.2. Ontologies

Ontologies are a methodology for standardizing terminology in a computable fashion to support the creation of logical axioms between related terms. Prominent ontologies in the biomedical sciences include the Gene Ontology [28] and the Human Phenotype Ontology [29], with many others related to foods, chemicals, and diseases [30–32]. Knowledge graphs (KGs) are a method for representing knowledge such as ontology content and instance level data in a graph structure in which nodes and edges are explicitly connected via semantic relationships [33]. Because of their innate high dimensionality, data inquiries can be conducted using KGs. However, the dimensionality of KGs can be reduced through embedding so they can support other analytic methodologies [34]. In our investigation, we aligned heterogeneous data regarding health, environment, and internal exposures to ontology content for ingestion into a KG, which was subsequently embedded and analyzed using machine learning techniques.

## 2. Methods

### 2.1. Data sets

The primary data for this project came from the Personalized Environment and Genes Study (PEGS, formerly known as the Environmental Polymorphisms Registry) conducted by the National Institute of Environmental Health Sciences (NIEHS) [35,36], which includes data from three respondent surveys, the Health and Exposure (self-reported diseases and phenotypes), Internal Exposome (foods, medications, supplements, and ingested exposures), and External Exposome (environmental exposures) surveys. Survey respondents are adult (aged 18 years or more) residents of North Carolina recruited for voluntary participation through health providers or events such as health fairs. The data included in this investigation were collected between 2012 and 2020. PEGS data is available by request only from NIEHS. This investigation was approved and deemed research with no human subjects (Category 4 exemption) by the Oregon State University (IRB-2021–1207).

Additional publicly available data were included in this investigation. Agricultural Chemical Usage Program (ACUP) data from the United States Department of Agriculture (USDA) on fungicides, pesticides, and other chemicals applied to agricultural crops during 2016–2020 was included for all relevant questions in the PEGS data sets (for instance, data on chemicals applied to carrots was included as PEGS inquires about consumption of carrots). ACUP data were not included if there was no related PEGS question, and not all PEGS questions about diet had related ACUP data (for example, consumption of combination foods such as hamburgers or foods without crop components, such as meat). Nutrient data for Foundation Foods from the USDA Food Data Central (FDC) was included when available with references to the FoodOn ontology [32]. This allowed for direct mapping to the selected ontology alignment (for instance, a survey question on intake of cottage cheese mapped to FOODON:03303720; and ‘cottage cheese (lowfat)’ mapped to FDC ID: 328,841 and FDC nutritional content for ‘Cheese, cottage, lowfat, 2 % milkfat’).

### 2.2. Knowledge graph data preparation

Combined, the PEGS surveys comprise 1842 questions. We assessed the survey questions for ontology alignment based on existing ontology content and complexity of the survey question as well as the primary topic area. We focused on questions related to diseases, phenotypes, dietary exposures, and environmental exposures. We then aligned feasible survey questions of interest (n = 341, with 135 from the External Exposure Survey, 131 from the Internal Exposure Survey, and 75 from the Health and Exposure Survey) to ontology terminology. An ontology curator (author LC) manually reviewed the data to map the PEGS survey questions to the coordinating ontology content. Free-response components of the PEGS surveys and other data sets, including USDA ACUP data, were mapped to ontology terms using semi-automated curation with OntoRunNER [37], followed by supplemental manual review by the curator. The ‘survey question label’ selected for free response questions was assigned the mapped ontology term value of the response due to the list aggregation used to process data via OntoRunNER. When necessary, we requested new ontology terms in efforts to support the mappings needed for this data alignment. Primary requests were made to the Food Ontology (FoodOn) [32] and the Environmental Conditions, Treatments, and Exposures Ontology (ECTO) [38].

### 2.3. Creating a KG

**Fig. 1.**
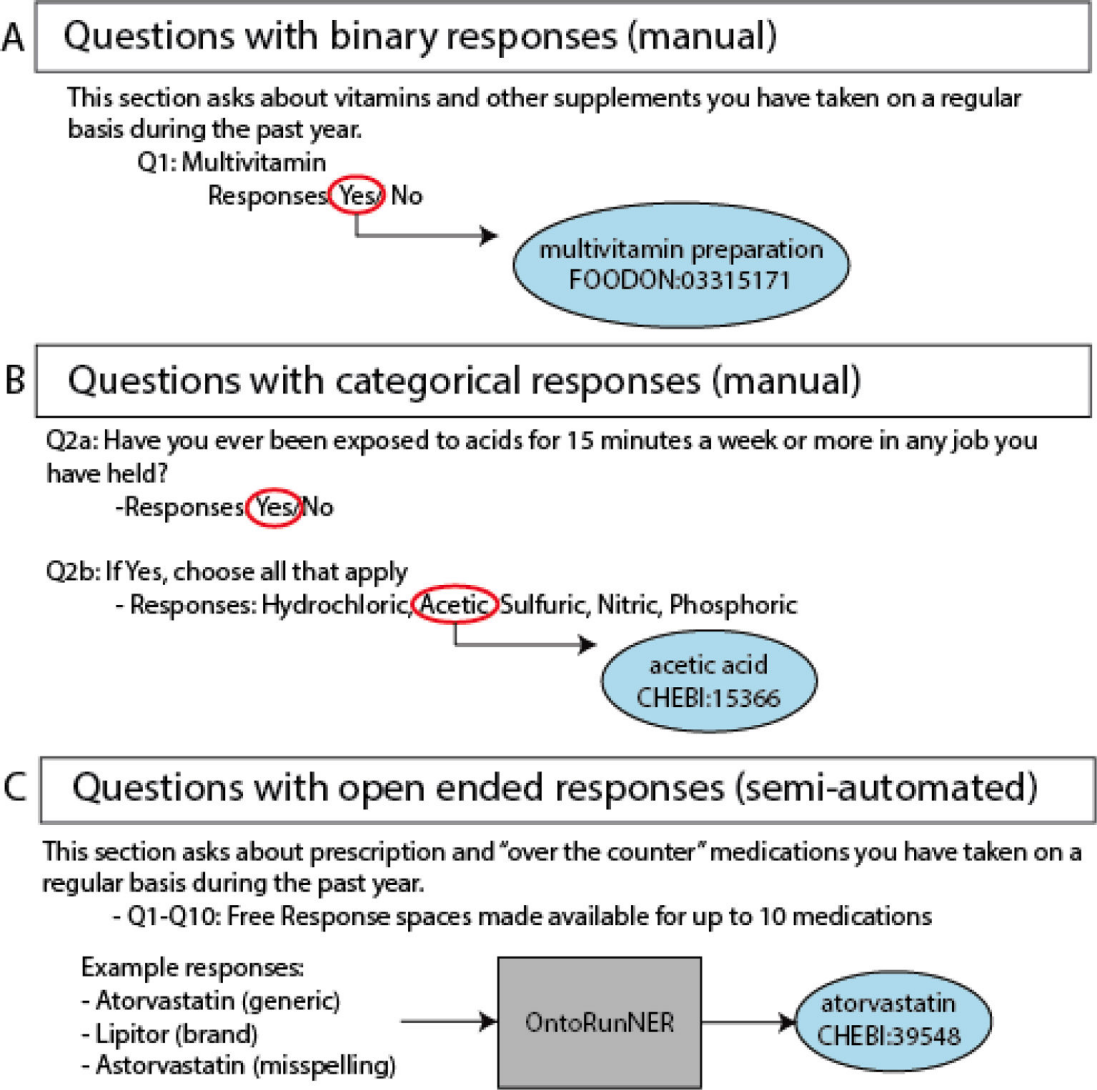
Translating survey questions to ontology content. In efforts to coordinate PEGS survey questions with ontology content, a combination of manual and semi-automated mappings was conducted. For questions with binary or categorical, finite responses, manual curation was used to align a single ontology term to the question (binary) or to each variable response option (categorical) (Fig. 1A/B). For free response questions, the named entity recognition tool, OntoRuNER was used to create mappings to ontology terms for unique answer fields (Fig. 1C). Ontology abbreviations: FOODON, Food Ontology. CHEBI, Chemical Entities of Biological Interest.

We created the KG for this project with an extract, transform, load (ETL) pipeline constructed using the Knowledge Graph Hub project KG-template [39]. The KG-template offers a skeleton structure of data download, transformation, and merge scripts that we customized for this project. This pipeline was developed using Python (Version 3.90.10) and Koza [40], a data transformation framework constructed by the Monarch Initiative. Transformations included the alignment of self-reported data for questions of interest with the ontology mappings generated manually or semi-automatically as described in Fig. 1. Code used for KG development is available at our GitHub repository(41).

**Fig. 2.**
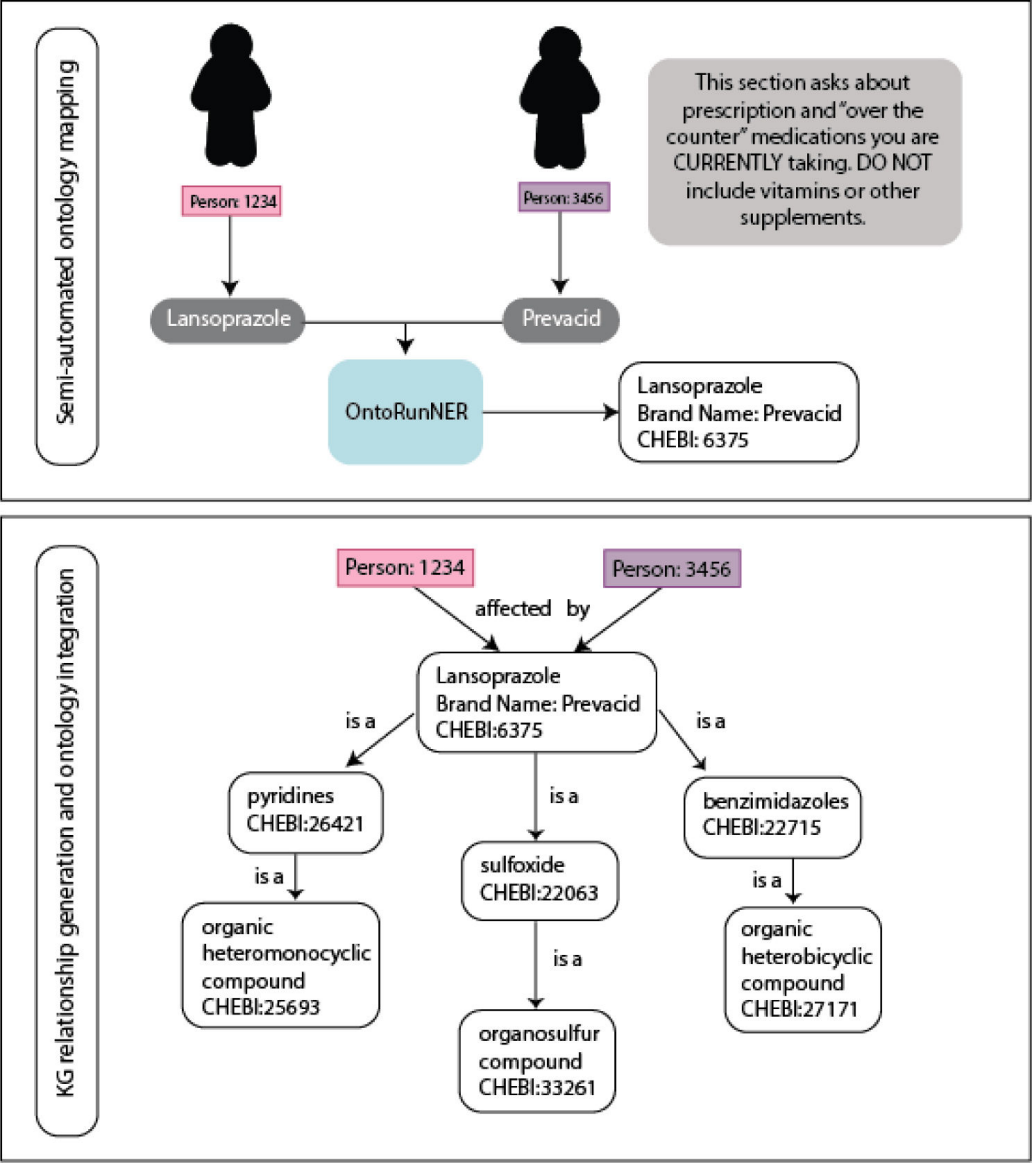
Coordinating respondent data to ontology content. Following completion of a survey question, the responses are used to generate an appropriate mapping of the response to an ontology term. During this process, nodes are established for each respondent as well as each positively answered survey question. Only unique nodes are generated, meaning only one node is created for each respondent and each survey question. Ontology terms have a corresponding hierarchy within the ontology that is also coordinated to the survey question and response. Unique “transformation” steps for each question type (for example, medication, environmental exposure, disease) are used to then create a three-part relationship including a subject, predicate, and object. As seen in this example question regarding medication usage, following a response of “lansoprazole”, Person:1234 had their response mapped using the semi-automated OntoRunNER tool to the appropriate ontology term and then the transformation step created a relationship result of “Person:1234 affected by Lansoprazole (CHEBI:6375)”. Given lansoprazole is contained within a hierarchy in the Chemical Entities of Biological Interest ontology, it is subsequently associated with a variety of terms in the taxonomy. As ontology content that is identified to have the same label, or shared synonyms will be mapped to the same node within a KG, a positive response of lansoprazole usage by the brand name Prevacid, similarly allows for the resulting relationship of “Person:3456 affected by lansoprazole”.

We conducted each data transformation (for instance, disease, phenotype, medication, food) with a unique script that asserted the correct “predicate” (for example, the phenotype transform created assertions such as ‘Person:1234’ ‘has phenotype’ ‘uterine leiomyoma’). We followed this process for all PEGS data and all supplemental data on food, chemical usage, and nutrient content. Fig. 2 provides an example of the full mapping and transformation process, in which reusable nodes were generated for a respondent’s unique ID and their survey responses. In turn, all questions answered by a respondent were mapped to the same respondent node using their ID. Similarly, all respondents who answered the same question were mapped to the same question response node. In addition to the transformed respondent data, the full contents of relevant ontologies (Human Phenotype Ontology (HPO), Mondo Disease Ontology (Mondo), Medical Actions Ontology (MAxO), Gene Ontology (GO), Environment Ontology (ENVO), Chemical Entities of Biological Interest (ChEBI), ECTO, and FoodOn) were merged to create the KG. Within the KG structure, each ontology term or survey participant was considered a “node”, with all relationships between each node considered an “edge”.

### 2.4. Embedding the KG

As with many KGs, the KG for this project was a high-dimensional object with a large number of nodes and edges, making it less amenable to machine learning. Lower-dimension forms of a KG allow for improved generalization of knowledge, as the latent representation places dissimilar nodes farther away from one another and nodes with greater similarity closer to each other. To reduce the dimensionality of the KG in preparation for machine-learning techniques, we embedded the KG using Graph Representation leArning, Predictions and Evaluation (GRAPE) [41] and its embedding library. We used only the largest component of the KG, which eliminated data from 691 (7.1 %) survey respondents due to insufficient data. The generated embedded representations included ontology terms, exposures, clinical variables, FRDs, and respondents. As such, the resulting representations embedded the topological relationships between the different types of entities populating the KG in a vectorial space. Additional details can be found in the Supplemental Methods.

**Fig. 3.**
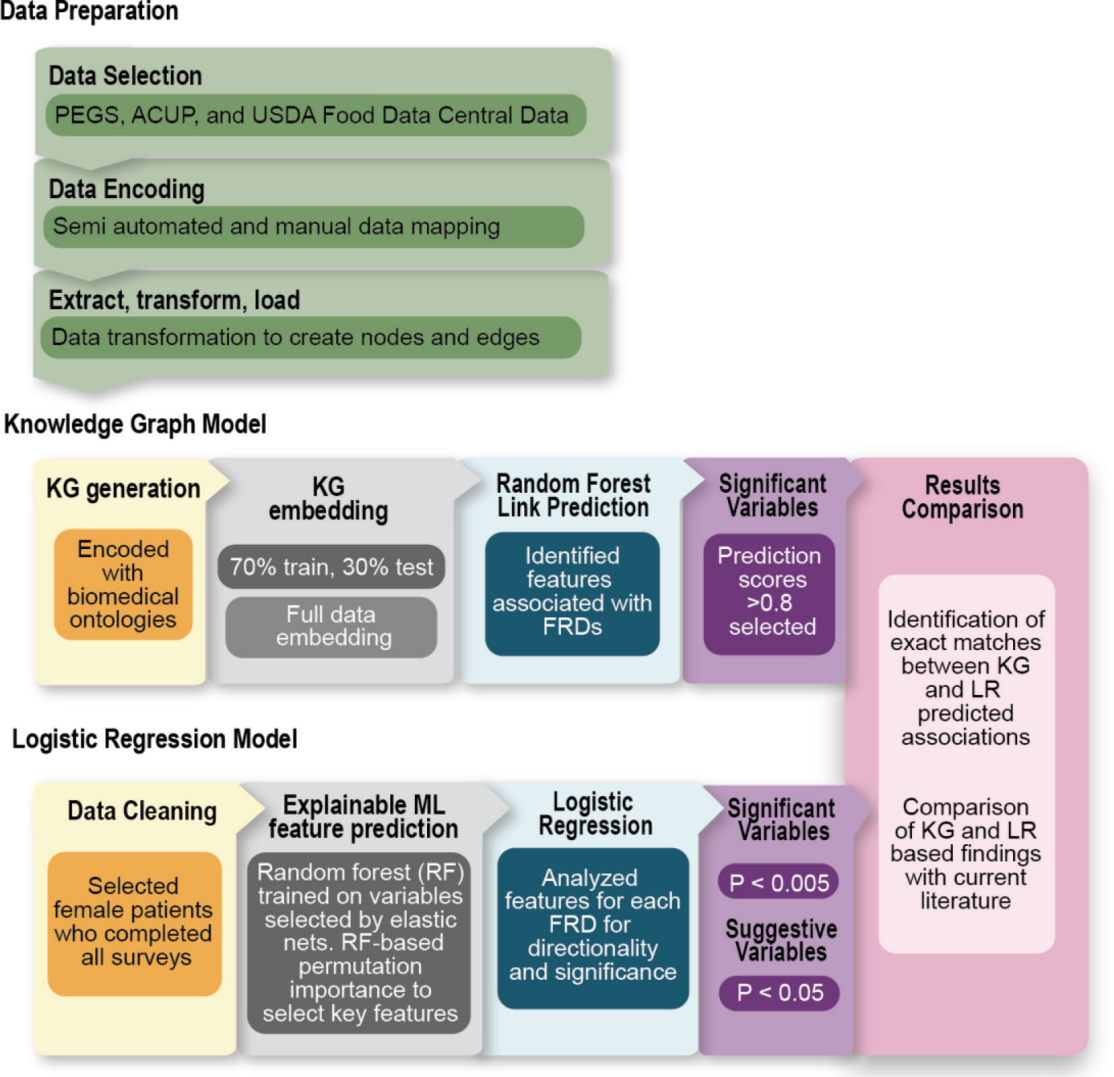
Computational methods overview. Starting with data preparation, our pipeline of data selection and encoding using biomedical ontologies harmonized our data for the transformations necessary to develop nodes and edges to construct our knowledge graph and logistic regression models. Two comparative analytical approaches were used to evaluate the Personal Environment and Genes Study (PEGS) survey data regarding internal and external exposures and personal health along with the Agricultural and Chemical Use Program (ACUP) and USDA Food Data Central data. The KG model included encoding all survey data with biomedical ontology content and creation of a KG structure, followed by embedding of the KG to create a low dimensional format for use in the random forest model to assess predicted links between FRDs of interest and exposures or health variables. The comparison logistic regression analysis system supported data interpretation by including 1) data cleaning, 2) application of elastic nets to initially select the most discriminative variables and improve regularization, 3) an explainable random-forest analysis that uses permutation-based feature importance to select important associations between exposures, health conditions, and FRDs, and 4) logistic regression to evaluate significance and directionality (interpretability) of the extracted associations.

For the following machine learning methods, we generated two edge-embedding versions, a training embedding and a full data embedding. The training embedding included a ‘Training’ portion comprising 70 % of the graph and a ‘Test’ portion comprising the remaining 30 %. We created the test portion by selecting and holding out edges that, when removed from the full embedding, did not create a new component and thus kept the primary component of the graph intact. This avoided a biased estimation of the edge prediction results for the test set (see the GRAPE github repository for a full description of the method [42]). Edges in the training set were not specifically selected as “positive” responses (for example, edges documenting an FRD-variable relationship), in efforts to train the model for edge prediction based on the entire topology of the graph. The full embedding included all available data. Fig. 3 summarizes the analytical methods.

### 2.5. Machine learning analyses

Random forests (RF) [43] are machine-learning classifiers used for computing medical predictions due to their inherent explainability and interpretability and the availability of methods (although preliminary) to convert them into a checklist of rules [44,45].

Our primary machine-learning task was applied to the KG we created, generating link predictions between variables (for example, food, nutrient, environmental exposure, disease, phenotype) and the FDRs of interest. We then trained an RF model (501 trees, 15 maximum depth) using the embeddings of the training data (with holdouts). The standard machine-learning performance metrics indicated the model was trained successfully and suitable for our analysis (area under the receiver operating characteristic (AUROC) = 0.915 for the ‘Test’ portion of the training data). To produce actionable results, we then retrained the model on the full dataset to obtain a set of predicted links between the FRDs and other variables. In the output, predicted links were represented by two node values—the “source” (independent variable) and “destination” (dependent variable) nodes of the link—and a “prediction” score indicating the strength of the predicted link between the two nodes. Utilizing the full graph embedding, we selected prediction outcomes from the model that included an FRD (for example, endometriosis, ovarian cysts, uterine fibroids) as the “source” and the resulting “destination”. We retained pairs with a prediction score > 0.8, resulting in a list of predicted variables for each FRD of interest.

### 2.6. Logistic regression analysis

For additional comparison of our KG findings, we conducted a secondary analysis using elastic nets, RFs, and logistic regression models to provide feature explanations (in terms of feature importance in prediction) and interpretations (in terms of the directionality of risk scores associated with each feature). We conducted this analysis in R, version 4.20.2. We cleaned the primary PEGS data on health conditions and internal and external exposures to include female participants only. We then excluded participants who did not complete all three surveys to improve data quality, given the lower response rates to the Internal and External Exposure Surveys versus the Health and Exposure Survey. For the regression analysis, we utilized only survey questions that aligned with the KG analysis (see KG Data Preparation) to maintain consistency and enable comparison. We imputed missing data using the missForest algorithm, which has exhibited superior performance in previous work [44,46].

To select the features with the strongest relationships with the FRDs of interest, we leveraged an explainable machine-learning technique [47], to account for the class imbalance affecting the FRD datasets and to produce both importance scores and their directionality concerning the risk of disease. We developed a model that applied a first step of supervised feature selection on the training set and then selected features used to train an RF classifier. The model then computed permutation-based feature importance scores based on the RF classifier that were used to select the most important variables for FRD prediction. Features regarded as important by an RF are not characterized by directionality and magnitude, which is important for a medical context [48]. To assess these characteristics, we then trained logistic regression classifiers, whose learned odds ratios and *P* values indicate the significance and directionality of risk scores. We ran the model three times, each time utilizing a different FRD as the primary outcome. We adjusted the *P* values obtained in the logistic regression analyses for endometriosis, ovarian cysts, and uterine fibroids using Bonferroni correction to account for the family-wise false discovery rate (FDR).

Based on the KG and logistic regression model results, we identified the most influential features for each FRD. We compared both the KG and logistic regression outputs for exact matches for each FRD. Details of additional methods can be found in the Supplemental Methods and our code can be found on GitHub [49].

## 3. Results

A total of 16,039 surveys were completed (External Exposome = 3579, Internal Exposome = 3034, Health and Exposure = 9426) by 9765 unique individuals, including 2773 individuals who completed all three surveys. In the study population, there was reported prevalence of 7 % for endometriosis, 15 % for uterine fibroids, and 13 % for ovarian cysts. Translation keys for all survey questions of interest and their coordinating ontology content, including OntoRunNER generated mappings, can be found in Supplementary Table 1A–D (Supp Table 1D is also available on github [50]). The majority of survey respondents were female, with an average age between 49.9 and 54 years depending on the survey (Table 1). Further information such as race/ethnicity, pregnancy history, age at menarche, and health care access level were not available in this dataset.

**Fig. 4.**
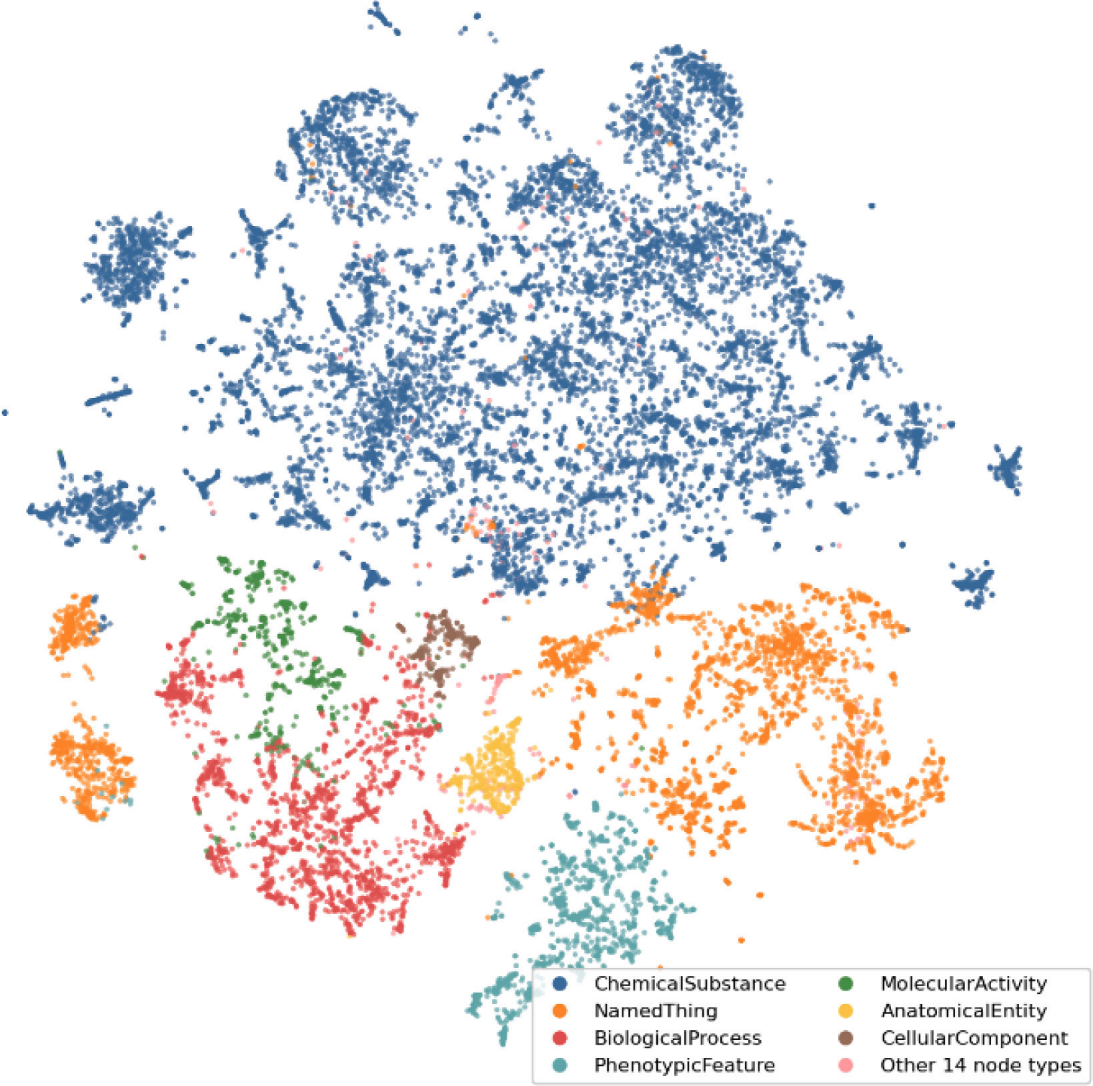
KG visualization. t-SNE visualization of the embeddings computed for the largest connected component in the KG. The node embeddings have been computed by using the DeepWalk algorithm followed by a Skipgram model, as implemented in the GRAPE library. The plot displays the variety of node types represented in the graph, where each node is represented by a dot and nodes with the same type are characterized by the same color. This visual serves as a preliminary assessment tool for the KG, showcasing how well the graph can decipher and cluster (conceptually and semantically) similar node types.

The KG created for this project has 308.60 K heterogeneous nodes and 696.68 K edges in total. The graph contains 28.44 K connected components (of which 28.41 K are disconnected nodes), with the largest one containing 280.03 K nodes and the smallest one containing a single node. Fig. 4 shows the resulting full graph embedding after selecting for the largest connected component in the graph.

We identified a list of significant (P < 0.005) and suggestive (P < 0.05) variable features from the logistic regression analyses and predicted significant findings from the KG (prediction score > 0.8). All survey labels were coded for a “Yes” response to the question, indicating the presence of an exposure or condition. Table 2A–C shows the significant (P < 0.005) and suggestive features (P < 0.05) identified from logistic regression. Significant or suggestive features from both analyses are indicated in bold in Tables 2A–C. Supplemental Tables 2A–C provide a full list of variables identified from logistic regression. A full list of variables identified as part of the KG link prediction methodology can be found in Supplemental Table 3 (Supp Table 3 is also available on Github [51]).

Table 2A–C. Significant and suggestive features identified via logistic regression. Variables that are direct matches in the KG results are displayed in bold. Unreported Mean Variance Inflation Factor (VIF) scores indicate inadequate information available to calculate the score.

## 4. Discussion

Our work developing a KG with survey-based data and conducting machine learning to predict variables associated with FRDs is the first of its kind. The logistic regression model we developed for comparison supports our findings using this novel approach. Comparing the logistic regression and KG models resulted in numerous exact matches for medical conditions and procedures, environmental exposures, medications, and dietary exposures for the considered FRDs. Endometriosis and ovarian cysts had suggestive associations with other gynecological conditions and procedures. Positive responses to questions regarding hysterectomy, ovary removal, and ovarian cysts were all suggestively associated with endometriosis. A possible explanation for the procedure associations is that ovary removal and hysterectomy are offered as endometriosis treatment options when other therapies have been unsuccessful [52,53]. However, the timing of disease onset and medical procedures in this dataset was unavailable. Endometriosis can present as an ovarian endometrioma, an endometriotic cyst in the ovary [54], which may be related to the suggestive endometriosis and ovarian cyst association identified. It is important to note that screening for any of these gynecological conditions may contribute to the identification of another gynecological comorbidity due to increased potential for detection.

Use of duloxetine had a suggestive association with uterine fibroids in this study. Duloxetine is a medication primarily used for treatment of major depressive disorder, generalized anxiety disorder, chronic musculoskeletal pain, and fibromyalgia [55]. While duloxetine does not have a documented relationship with FRDs in current literature, there is a strong association between depression and mental health concerns in individuals with FRDs. Individuals with uterine fibroids have been documented to experience higher rates of depression and anxiety compared to controls, particularly amongst individuals who experience pain symptoms or who have undergone a hysterectomy [56]. Given the increased prevalence of mental health conditions amongst individuals with FRDs, individuals with these conditions may be more likely to take antidepressants or similar medications which may be related to this finding.

Omeprazole use was significantly associated with increased odds of uterine fibroids. Omeprazole is a proton pump inhibitor, used to treat gastroesophageal reflux disease (GERD), ulcers, and other conditions characterized by excessive stomach acid [57]. Omeprazole has no reported side effects related to uterine fibroid development, but bulk-related symptoms may present due to uterine fibroids as the enlarged fibroids can distort the abdominal anatomy and cause abdominal bloating and pressure [58]. Uterine fibroids have been denoted as an associated disorder for individuals with Barrett’s esophagus, a gastrointestinal complication of GERD [59,60].

We identified multiple potential associations between diet and FRDs. Tofu consumption was suggestively associated with decreased odds of endometriosis. Tofu, a processed soybean curd, is often studied for its health benefits related to its high isoflavone content [61]. Isoflavones are of interest given their known antioxidant properties [62]. It is hypothesized that excessive inflammation observed with endometriosis may be mitigated through isoflavone exposure [62,63]. Supporting the suggestive association of the present study, prior work has reported an inverse relationship between urinary isoflavone concentration and severe endometriosis [64]. However, a set of case studies investigating excessive soy consumption found high soy intake to be related to dysmenorrhea, endometriosis, and uterine fibroids [65]. Because of the higher rates of soy consumption among Asian individuals compared to other groups [66], it is notable that prevalence of endometriosis is higher in Asian populations than in other racial groups [67,68]. However, data on race were unavailable for analysis. Notably, soy isoflavones are also phytoestrogens, given their ability to bind to estrogen receptors and contribute to estrogenic activity in humans [62]. Isoflavones have been denoted as potential endocrine disruptors, however these long-term mechanistic effects are not fully elucidated [61]. While our results are inconclusive, further research evaluating soy consumption and endometriosis may be helpful for guidance on prevention and management.

A suggestive association was also identified for carrot consumption and decreased odds of endometriosis. Consumption of fruits and vegetables has been identified as protective against endometriosis, potentially due to the anti-inflammatory properties of dietary components, including vitamins C and E [69,70]. Carrots contain high levels of antioxidant carotenoids, which may reduce the inflammatory responses that occur in individuals with endometriosis [71]. The effects of carrot consumption are inconsistent in the literature, with multiple investigations reporting no significant associations between carrots and endometriosis [72,73]. Further exploratory work is needed for all potential dietary relationships with FRDs, including study designs which can include food quantities, as that was a limitation of this study design.

By utilizing a novel KG methodology and comparing the results with those from a traditional logistic regression model, we generated and corroborated multiple hypotheses of the effects of modifiable lifestyle factors on FRDs. The KG method presented here is an effective hypothesis-generation strategy, but the results should not be construed as causal as in other survey-based methodologies. Due to a lack of temporality information regarding exposures and condition onset, hypotheses generated from these associations should be investigated bidirectionally to best interpret how the variables interact.

The logistic regression approach indicated positive or negative associations for survey variables, which cannot be calculated using existing KG methods. The KG model identified an unranked list of predicted significant factors that require further assessment to identify variables of interest. Given the novelty of applying the KG method in survey-based data, its successful application in the present work showcases the potential of computational survey investigations using biomedical ontologies. Collecting data with ontology alignment in mind or retroactively performing ontology alignment for secondary data analysis will provide opportunities to apply KG study designs for hypothesis generation.

## 5. Limitations

This work has limitations due to the nature of the PEGS dataset, namely the North Carolina-specific population and the lower percentage of individuals with FRDs compared to national prevalence estimates. While this investigation was a secondary data analysis and did not involve design or collection of PEGS survey data, future investigations should include a more geographically diverse sample population for greater generalizability of study findings. Additionally, the dataset lacks information on temporality. PEGS participants are asked to describe their current eating habits, past and current exposures, and whether they have been diagnosed with an FRD. Given the lack of context for when onset of a condition occurred, it is difficult to identify the true impact of diet or environmental exposures, as they may have occurred before or after symptom presentation and disease diagnosis. Use of a survey design that includes temporality questions and collects information on gynecological history, demographics, and other potential confounders may improve the interpretation of findings.

Of note, our investigation used a binary variable of food consumption for individuals to indicate that they either do or do not consume a particular food. This approach was consistent for all food exposures, with no distinctions made between low and high consumption. Given the potentially wide range of consumption levels, this binary approach reduces the ability to decipher the impacts of dietary factors using the KG model. Binning data into “low”, “medium”, or “high” consumption levels (for example, “low” consumers eat apples 0–1 times per week) should be considered for future KG based investigations, to improve data output granularity. Further, our named entity recognition approach to mapping string responses to survey questions can be improved by grouping similar medications (for instance, regular versus extended-release formats). Additionally, machine learning approaches that consider specific values for dietary intake (for example, the number of apples consumed per week) when creating link predictions in a KG model would greatly benefit future nutrition investigations for hypothesis generation and potential future causally predictive works.

The performance of our KG model resulted in a substantial list of findings, many with similarly high prediction scores. While edge prediction provides prediction values between 0 and 1, equally ranked results make prioritization for hypothesis generation challenging. As such, efforts should be made to improve the prioritization of KG findings to enable hypothesis development.

While areas for improvement exist in this study design, we identified multiple predicted variables, including modifiable lifestyle factors, for FRD. Additional results, including those resulting exclusively from KG analysis, may result in meaningful hypotheses in future investigations of FRDs.

## 6. Conclusion

FRDs are highly impactful conditions for women globally, and there is a need to identify modifiable factors associated with these disorders. Limited investigations using ontologies or KG structures for investigations of FRDs have been conducted, and most existing studies have not accounted for modifiable lifestyle factors such as diet and environmental exposures. Using KG and logistic regression approaches, we identified a variety of potential intervention points for FRDs that can be pursued in future work. Because they are based on open-source, biomedical ontologies and computational resources, the novel methodologies used in this study can be repurposed for additional investigations.

### 6.1. Summary Table

- Computational analysis methods for nutrition and exposure survey data are limited, reducing their impact on treatments for conditions like FRDs.
- Although previous investigations evaluate FRD mechanisms and interventions, there are significant gaps in knowledge regarding modifiable lifestyle risk factors.
- This investigation harmonizes nutrition and exposure data with biomedical ontologies for FRD knowledge graph (KG) creation.
- KG analysis via a graph-representation-learning (GRL) model identifies variables which may significantly impact FRDs; these results are compared with a classic explainable AI technique, where the significance and risk of crucial variables identified via random forest-based, permutation-importance analysis are assessed by logistic regression.

## Supplementary Material

Supplemental methods

Supplemental Tables

## Figures and Tables

**Table 1:** Demographics for PEGS survey data.

	Health and Exposure (n = 9426)	External Exposures (n = 3579)	Internal Exposures (n = 3034)

BMI (mean, % missingness)	28.1 (0.02)	28 (0.1)*	27.9 (0.09)*
Gender (% female, % missingness)	67.1 % (0)	69.5 % (0.1)*	69.6 % (0.09)*
Age (mean, % missingness)	49.9 years (0.01)	54 years (0)	53.9 years (0)

* Estimated value inferred from Health and Exposures data.

**Table 2A:** Endometriosis logistic regression analysis, p-values are Bonferroni adjusted.

Odds	95 % CI lower limit	95 % CI upper limit	Standard Error	Survey question topic	Class ID	p-value	Mean prevalence	Sd prevalence	Missingness rate	Mean VIF

**20.71**	**10.55**	**40.80**	**0.29**	**Migraine**	**HP:0002076**	**50.12E-04** *	**0.25**	**0.43**	**0.01**	**10.47**
60.84	20.37	230.46	0.58	Uterine polyps	MONDO:0006195	80.44E-04*	0.06	0.24	0.00	10.54
**0.70**	**0.55**	**0.90**	**0.13**	**Carrot consumption**	**ECTO:0070046**	**60.33E-03**	**20.83**	**10.02**	**0.02**	**10.79**
**20.91**	**10.33**	**60.47**	**0.40**	**Ovary removal**	**MAXO:0001067**	**70.99E-03**	**0.15**	**0.36**	**0.00**	**20.75**
**20.19**	**10.21**	**40.00**	**0.30**	**Ovarian cysts**	**HP:0000138**	**90.75E-03**	**0.22**	**0.41**	**0.00**	**10.63**
**20.44**	**10.22**	**40.94**	**0.36**	**Hysterectomy**	**MAXO:0001058**	**10.20E-02**	**0.20**	**0.40**	**0.00**	**20.32**
220.18	20.47	>50	10.24	Metoprolol succinate use	CHEBI:6905	10.23E-02	0.04	0.19	0.00	
**0.22**	**0.06**	**0.73**	**0.62**	**Osteoporosis**	**HP:0000939**	**10.35E-02**	**0.04**	**0.19**	**0.01**	**10.67**
**10.69**	**10.08**	**20.66**	**0.23**	**Pesticide Exposure**	**ECTO:0000530**	**20.21E-02**	**0.44**	**0.50**	**0.05**	**10.36**
**0.78**	**0.63**	**0.96**	**0.11**	**Dark chocolate consumption**	**ECTO:0070138**	**20.22E-02**	**20.39**	**10.12**	**0.02**	**10.53**
0.09	0.01	0.61	10.09	Metoprolol use	CHEBI:6904	30.04E-02	0.05	0.22	0.00	
50.95	10.36	420.05	0.84	Desiccated thyroid extract use	CHEBI:9584	30.29E-02	0.01	0.12	0.00	10.36
0.23	0.05	0.83	0.69	Occupational alcohol exposure	ECTO:9000026	30.36E-02	0.19	0.39	0.11	
40.41	10.13	20.13	0.72	Occupational isopropanol exposure	ECTO:9000099	40.02E-02	0.14	0.35	0.00	
**0.75**	**0.57**	**0.99**	**0.14**	**Tofu consumption**	**ECTO:0070185**	**40.20E-02**	**10.56**	**0.91**	**0.02**	**10.70**
**10.92**	**10.01**	**30.69**	**0.33**	**Thyroid disease**	**MONDO:0003240**	**40.71E-02**	**0.17**	**0.38**	**0.01**	**10.57**

* P-values < 0.005.

**Table 2B:** Uterine fibroid logistic regression analysis, p-values are Bonferroni adjusted.

Odds	95 % CI lower limit	95 % CI upper limit	Standard Error	Survey question topic	Class ID	p-value	Mean prevalence	Sd prevalence	Missingness rate	Mean VIF

40.20	20.58	60.94	0.25	Hysterectomy	MAXO:0001058	10.20E-08*	0.20	0.40	0.00	20.04
**50.01**	**20.47**	**10.94**	**0.38**	**Uterine polyps**	**MONDO:0006195**	**10.90E-05** *	**0.06**	**0.24**	**0.00**	**10.27**
**30.71**	**10.77**	**80.35**	**0.39**	**Omeprazole**	**CHEBI:7772**	**80.29E-04** *	**0.05**	**0.22**	**0.00**	**10.62**
10.69	10.16	20.49	0.20	Iron deficiency anemia	HP:0001891	70.04E-03	0.24	0.43	0.00	10.26
10.71	10.16	20.54	0.20	Hypertension	HP:0000822	70.27E-03	0.29	0.45	0.00	10.92
10.70	10.13	20.56	0.21	Menopause	GO:0042697	10.04E-02	0.50	0.50	0.01	20.10
10.69	10.12	20.54	0.21	Ovarian cysts	HP:0000138	10.19E-02	0.22	0.41	0.00	10.41
**10.22**	**10.04**	**10.43**	**0.08**	**Orange consumption**	**ECTO:0070029**	**10.37E-02**	**20.53**	**10.05**	**0.02**	**10.63**
**10.69**	**10.07**	**20.68**	**0.23**	**Magnesium supplementation**	**ECTO:9000210**	**20.46E-02**	**0.16**	**0.37**	**0.01**	**10.43**
**20.58**	**10.11**	**60.34**	**0.44**	**Kidney infection**	**HP:0012330**	**30.25E-02**	**0.05**	**0.22**	**0.01**	**10.30**
10.42	10.02	10.96	0.17	Vitamin D supplementation	ECTO:9000133	30.53E-02	0.51	0.50	0.01	10.55
**30.97**	**10.12**	**180.82**	**0.70**	**Duloxetine**	**CHEBI:36796**	**40.82E-02**	**0.01**	**0.12**	**0.00**	**10.40**
0.57	0.32	10.00	0.29	Gallbladder disease	MONDO:0005281	40.91E-02	0.10	0.31	0.01	10.42

* P-values < 0.005.

**Table 2C:** Ovarian cysts logistic regression analysis, p-values are Bonferroni adjusted.

Odds	95 % CI lower limit	95 % CI upper limit	Standard Error	Survey question topic	Class ID	p-value	Mean prevalence	Sd prevalence	Missingness rate	Mean VIF

40.17	20.30	70.75	0.31	Ovary removal	MAXO:0001067	30.92E-06*	0.15	0.36	0.00	
**10.31**	**10.12**	**10.54**	**0.08**	**Spinach consumption**	**ECTO:0070060**	**90.94E-04** *	**20.61**	**10.11**	**0.03**	**10.59**
**0.13**	**0.04**	**0.43**	**0.63**	**Occupational methanol exposure**	**ECTO:9000028**	**10.35E-03** *	**0.07**	**0.25**	**0.00**	
**30.14**	**10.58**	**60.66**	**0.36**	**Uterine polyps**	**MONDO:0006195**	**10.73E-03** *	**0.06**	**0.24**	**0.00**	**10.19**
0.42	0.23	0.72	0.29	Hysterectomy	MAXO:0001058	20.22E-03*	0.20	0.40	0.00	
40.03	10.62	110.68	0.50	Tylenol	CHEBI:46195	40.96E-03*	0.03	0.17	0.00	10.21
**0.80**	**0.68**	**0.93**	**0.08**	**Bell pepper consumption**	**ECTO:0070042**	**40.99E-03** *	**20.80**	**10.04**	**0.02**	**10.39**
**10.12**	**10.04**	**10.22**	**0.04**	**Coffee consumption**	**ECTO:0070134**	**50.11E-03**	**30.57**	**10.94**	**0.03**	**10.21**
10.22	10.05	10.41	0.07	Butter spread consumption	ECTO:0070013	70.84E-03	10.72	10.10	0.02	10.23
0.57	0.37	0.87	0.22	Menopause	GO:0042697	90.31E-03	0.50	0.50	0.01	
10.66	10.13	20.44	0.19	Migraine	HP:0002076	90.34E-03	0.25	0.43	0.01	10.21
0.77	0.63	0.94	0.10	Tofu consumption	ECTO:0070185	10.22E-02	10.56	0.91	0.02	10.45
**20.69**	**10.25**	**60.20**	**0.40**	**Bupropion**	**CHEBI:3219**	**10.46E-02**	**0.05**	**0.21**	**0.00**	**10.18**
**20.53**	**10.22**	**50.48**	**0.38**	**X-ray exposure**	**ECTO:8000046**	**10.49E-02**	**0.08**	**0.27**	**0.00**	**10.42**
40.19	10.40	150.67	0.60	Vitamin D supplement	CHEBI:27300	10.72E-02	0.03	0.16	0.00	10.22
**10.63**	**10.09**	**20.45**	**0.21**	**Uterine fibroids**	**HP:0000131**	**10.76E-02**	**0.24**	**0.43**	**0.00**	**10.26**
**30.13**	**10.17**	**80.67**	**0.51**	**Occupational isopropanol exposure**	**ECTO:9000099**	**20.47E-02**	**0.14**	**0.35**	**0.00**	
20.11	10.11	40.11	0.33	Kidney stones	HP:0000787	20.52E-02	0.07	0.26	0.01	10.22
**10.58**	**10.04**	**20.43**	**0.22**	**Probiotic consumption**	**ECTO:0070000**	**30.32E-02**	**0.19**	**0.39**	**0.01**	**10.33**
30.54	10.19	130.00	0.59	Desiccated thyroid extract use	CHEBI:9584	30.32E-02	0.01	0.12	0.00	10.20
**20.34**	**10.09**	**50.38**	**0.40**	**Fibromyalgia**	**MONDO:0005546**	**30.55E-02**	**0.05**	**0.22**	**0.01**	**10.30**
**0.84**	**0.71**	**0.99**	**0.09**	**Blueberry consumption**	**ECTO:0070025**	**30.70E-02**	**20.58**	**10.09**	**0.02**	**10.55**
**0.16**	**0.03**	**0.84**	**0.87**	**Occupational butanol exposure**	**ECTO:9000424**	**30.74E-02**	**0.01**	**0.11**	**0.00**	**10.17**
40.25	10.21	20.24	0.70	Occupational cleaning liquid exposure	ECTO:0500011	30.78E-02	0.29	0.45	0.05	
**20.85**	**10.09**	**80.18**	**0.51**	**Occupational chloroform exposure**	**ECTO:9000042**	**30.89E-02**	**0.06**	**0.23**	**0.00**	
80.66	10.50	1640.37	10.08	Tramadol	CHEBI:9648	40.62E-02	0.01	0.10	0.00	10.16

* P-values < 0.005.
