# Supplementary material for: Predicting nutrition and environmental factors associated with female reproductive disorders using a knowledge graph and random forests
Source: Int J Med Inform. Author manuscript; Available in PMC 2024 Jul 1. (PMC11188727; doi:10.1016/j.ijmedinf.2024.105461)
Supplement: Supplemental methods [file NIHMS1992545-supplement-MMC2.docx]

**SUPPLEMENTAL METHODS**

**OntoRunNER**

OntoRunNER is a named entity recognition (NER) tool that reads strings of written content, which is then compared to a designated term list to identify what content is an exact or close match to the term list provided. For this project, we used OntoRunNER to coordinate strings of data input primarily regarding chemicals (medications or agricultural chemicals) to Chemical Entities of Biological Interest (ChEBI) Ontology terms. OntoRunNER allowed for both exact matches of the primary ChEBI label as well as synonyms of ChEBI content. It also allowed the string and ontology term to be considered a “match” if there were four or fewer different characters between them. Following use of OntoRunNER, we hand-reviewed terminology mappings for accuracy, which included additional manual mapping creation for common misspellings (for instance, ‘aderall’ versus ‘adderall’). We excluded from the dataset any string that could not be confidently mapped using OntoRunNER or manual means in an effort to avoid introducing inaccuracies.

**Knowledge graph preparation for embedding**

To prepare the graph for embedding using GRAPE, we removed all disconnected (singleton) nodes that shared no edges with other nodes as they provided little to no information for our link prediction model. We then selected the largest connected-component of the graph, that is the subgraph of the knowledge graph containing the highest number of nodes and thus offering the greatest capacity to develop link predictions. Using only the largest component removed 7.1% of respondents (n = 691) from the final analysis. As these respondents were not included in the largest component, it is likely the data available from their survey responses was insufficient for informing significant link predictions.

We used the DeepWalkSkipGram approach to learn the latent representations of the nodes within this graph network. This approach uses the Deep Walk deep learning approach, where nodes within the graph are treated like words, and random walks between nodes can be taken to create sentences [[34]](https://paperpile.com/c/m5UM3T/Qj2d). The Skip-gram component includes inputting a single node and then contextualizing and classifying the word based on other words from the same sentence, allowing for projections of words coming both before and after the single word (node) of interest [[74]](https://paperpile.com/c/m5UM3T/hBNL). This approach to embedding allows for the creation of sentence structure using nodes and then the assessment of which nodes should be located near each other in the low-dimensional embedding visualization.

**Logistic regression analysis**

Logistic regression analysis was applied to those features regarded as important by an explainable AI approach where random forest classifiers (RFs) are applied in an unbiased way to extract the most important variables for prediction, that is those variables effectively impacting the discrimination between healthy and pathological subjects. While the unbiased application of RFs allows selecting the features that mostly relate to disease, the importance of such variables, as computed by RFs has no directionality. In other words, the RF-importance score does not provide clues about the variable being a risk factor or a protective factor for disease. To identify directionalities we therefore analyzed the important variables identified by RF through logistic regression.

RF analysis. To compute unbiased RF-importance scores, we trained and tested RF classifiers in an unbiased way, that is by applying a multiple holdout training and testing procedure, where we used 50 external stratified holdouts (train:test ratio 0.9:0.1). Each external training holdout was used to:

(1) apply a supervised feature selection algorithm (details in the next paragraph), which selected the most discriminative variables;

(2) train an RF classifier on the selected variables. To avoid overfitting, we trained the RF by balancing the samples used to choose each split (sampsize parameter in the randomForest function of the R package “randomForest”) and we optimized the RF parameters (number of trees for the RF and the number “mtry” of variables considered to define each split) by a grid search on 100 internal rebalanced holdouts (train:test ratio = 0.9:0.1) to maximize the area under the precision-recall curve, which is the most appropriate evaluation measure in the case of imbalanced classes.

(3) compute the permutation-importance score, which measures the variable importance as the mean decrease in accuracy when the variable is permuted.

The trained RF was then applied to the (left-out) test set to validate the RF approach. The variables that, on the average of all the external holdouts, had a permutation-importance score greater than zero were chosen as the most important features, whose directionality was then assessed by logistic regression.

Supervised feature selection applied on the training set. Supervised feature selection was applied on the training set, by using 50 internal stratified holdouts (train:test ratio = 0.9:0.1) to improve robustness. More precisely, the feature selection algorithm was iteratively applied on the 50 training holdouts; at the end of the iterations, the features selected most of the time across the holdouts were considered as the most discriminative features to be selected. To choose among the several available feature selection algorithms, we ran preliminary experiments to compare univariate feature selection techniques (where variables showing significant correlation with the label were selected), Boruta feature selection [[75]](https://paperpile.com/c/m5UM3T/XOhj), permutation-based RF importance, and elastic nets (where the value of the elastic-net regularization parameter $\lambda$ is set via internal five-fold cross-validation and the $\alpha$ parameter balancing the amount of lasso and ridge constraints is set to 0.5) [[76]](https://paperpile.com/c/m5UM3T/BpdL). Given the comparable preliminary results, we opted for elastic nets due to their higher regularization capability, which results in a lower number of selected features.

Next, for the directionality of scores, the important variables were used to train *logistic regression classifiers.* Considering how rare events resulting in highly imbalanced datasets may cause sharp logistic regression underestimates [[48]](https://paperpile.com/c/m5UM3T/iqUZ), we ran logistic regression on 100 holdouts rebalanced by undersampling. We averaged the results of the 100 iterations (odds and *P* values) to get the final estimates. We also calculated the variance inflation factor (VIF) and mean prevalence for each variable. These values are reported with the full logistic regression results in Supplemental Tables 2A-C. Of note, variables with a large VIF (>4) or a low mean prevalence score (0.001) may not be reliable regression outcomes due to colinearity or lack of sufficient data to determine the influence of a variable on the outcomes of interest.
